# Controlling nutritional status score is associated with renal progression, cardiovascular events, and all-cause mortality in biopsy-proved diabetic kidney disease

**DOI:** 10.3389/fphys.2023.1231448

**Published:** 2023-08-07

**Authors:** Qingyu Huo, Ting He, Jiachuan Xiong, Jinghong Zhao

**Affiliations:** Department of Nephrology, The Key Laboratory for the Prevention and Treatment of Chronic Kidney Disease of Chongqing, Chongqing Clinical Research Center of Kidney and Urology Diseases, Xinqiao Hospital, Army Medical University (Third Military Medical University), Chongqing, China

**Keywords:** diabetic kidney disease, controlling nutritional status (CONUT) score, end-stage renal disease, cardiovascular events, mortality

## Abstract

**Background:** The Controlled Nutritional Status (CONUT) score, calculated from albumin, total cholesterol, and lymphocyte count, is a useful indicator for immune-nutritional assessment and is associated with the prognosis of various diseases. However, its relationship with renal outcomes, cardiovascular disease (CVD), and all-cause mortality in patients with diabetic kidney disease is unclear.

**Methods:** This retrospective single-center study enrolled 336 patients with biopsy-confirmed diabetic kidney disease from August 2009 to December 2018. The outcomes were progression to end-stage renal disease (ESRD), CVD events, and death. Univariate and multivariate Cox regression analyses were performed to estimate the association between confounding factors and outcomes. The Kaplan-Meier curve was used to compare the outcomes of the patients according to the median CONUT score. The area under the curve (AUC) evaluated with time-dependent receiver operating characteristics was used to test discriminative power of COUNT score.

**Results:** During a median follow-up period of 5.1 years. The Kaplan-Meier analysis showed that patients in the high CONUT group (CONUT score > 3) had a significantly higher incidence of ESRD, CVD events, and all-cause mortality than those in the low CONUT group (CONUT score ≤ 3). The multivariate COX regression analysis indicated that, The CONUT score was an independent predictor of ESRD (hazards ration [HR] = 1.129, 95% confidence interval [CI] 1.037-1.228, *p* = 0.005), CVD events (HR = 1.159, 95% CI 1.057-1.271, *p* = 0.002), and all-cause mortality (HR = 1.299, 95% CI 1.143-1.478, *p* < 0.001).

**Conclusion:** The CONUT score is an independent risk factor for ESRD, CVD events, and overall death in patients with diabetic kidney disease.

## 1 Introduction

Diabetic kidney disease (DKD) is a common complication of diabetes that affects up to 50% of diabetic patients and is the leading cause of end-stage renal disease (ESRD) globally. It is also associated with a significant increase in cardiovascular disease (CVD) morbidity and mortality, making it a major public health concern (Selby and Taal, 2020). Traditional risk factors such as metabolic abnormalities and hemodynamic stress are well-known contributors to these unfavorable clinical outcomes (Giunti et al., 2006). Renin-angiotensin system (RAS) inhibitors, sodium-glucose cotransporter-2 inhibitors (SGLT-2is), glucagon-like peptide-1 receptor agonists (GLP-1RAs), and mineralocorticoid receptor antagonist (MRA) have been proven to effectively mitigate the impact of these risk factors on diabetic kidney disease patients (Yang et al., 2022; Filippatos et al., 2023; Giglio et al., 2023). However, a growing body of research indicates that chronic inflammation, immune dysregulation, and nutritional status also play crucial roles in the progression of diabetic kidney disease (Yang and Mou, 2017; Zhang et al., 2022; Liu et al., 2023; Zhang et al., 2023). As such, early identification and management of these non-traditional risk factors may be critical for optimizing the prognosis of diabetic kidney disease.

The Controlled Nutritional Status (CONUT) score is an immune-nutritional index developed by Ulibari et al. in 2005 for early monitoring of the nutritional status of hospitalized patients. It is calculated based on the serum albumin level, total cholesterol concentration, and total lymphocyte count (Ignacio de Ulíbarri et al., 2005). These three indicators represent information on nutritional status, immune function, and lipid metabolism, respectively, and can be obtained through routine blood analysis, making the CONUT score feasible and reproducible. Increasingly, studies have shown that the CONUT score can predict the prognosis of solid tumors and hematologic diseases (Xue et al., 2021; Zhou et al., 2021; Liu et al., 2022). Additionally, the CONUT score is an independent predictor of all-cause mortality and CVD in patients with coronary artery disease (Arero et al., 2022). In kidney diseases, the CONUT score is a strong predictor of CVD morbidity and all-cause mortality in peritoneal dialysis patients (Yang et al., 2020; Zhou et al., 2020), and is independently associated with previous CVD in patients with chronic kidney disease (Tsuda et al., 2023). Furthermore, the CONUT score has been shown to predict all-cause mortality in patients on hemodialysis (Takagi et al., 2022), and is significantly associated with disease activity in lupus nephropathy (Ahn et al., 2019). However, the potential role and prognosis of the CONUT score in diabetic kidney disease are rarely reported.

In this study, we aim to determine the prognostic value of CONUT scores in individuals with diabetic kidney disease for ESRD, CVD, and all-cause mortality.

## 2 Materials and methods

### 2.1 Study Design

This retrospective study included 336 patients with biopsy-confirmed DKD from Xinqiao Hospital of the Army Medical University in China between August 2009 and December 2018. DKD was diagnosed based on criteria established by the Renal Pathology Society in 2010 (Tervaert et al., 2010). All participants were followed up from the screening date until 31 December 2021, or until their death. The study protocol was approved by the ethical committee of Xinqiao Hospital (No. 2018-006-02).

**FIGURE 1:**
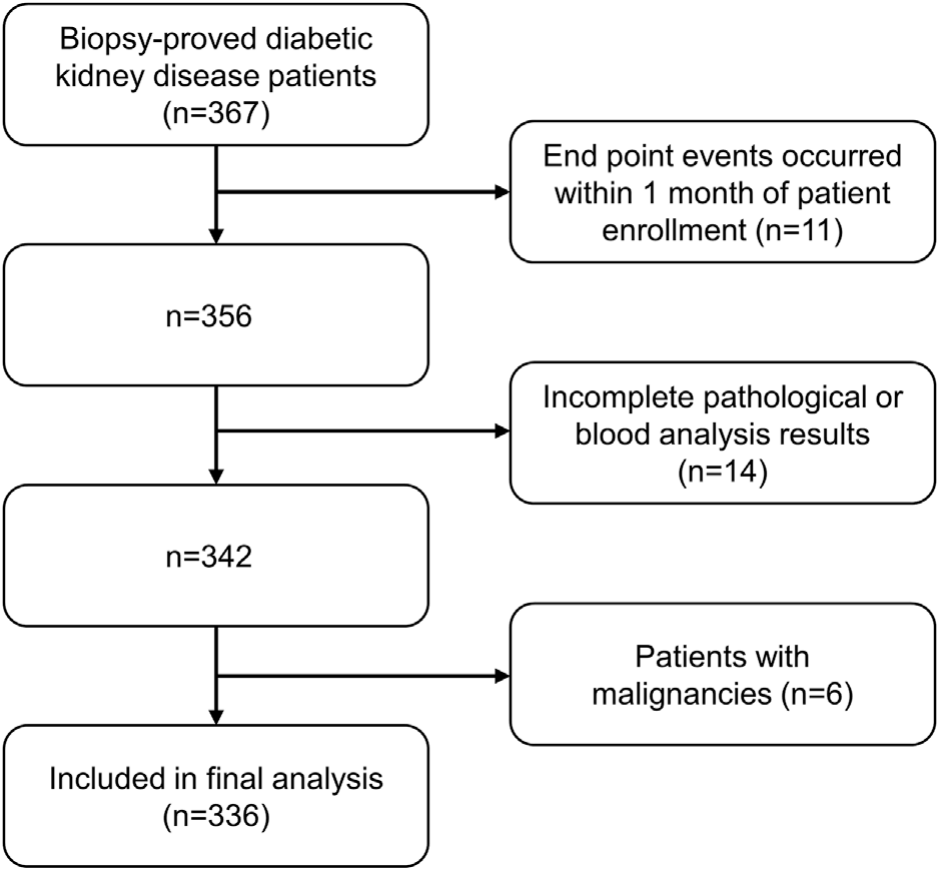
Flowchart of included patients in this study. ESRD: end-stage renal disease. CVD: Cardiovascular disease.

Inclusion criteria were: 1) biopsy-confirmed diabetic kidney disease (including pure diabetic nephropathy and diabetic nephropathy combined with non-diabetic nephropathy); 2) adults aged 18 years or older; 3) complete medical information and follow-up data. Exclusion criteria were: 1) ESRD, CVD events, or all-cause death took place within 1 month of follow-up after enrollment; 2) patients with incomplete pathological information or blood routine examination; 3) patients with malignancies (e.g., breast, lung, gastrointestinal, hematologic cancers). (Figure 1).

### 2.2 Data collection

We extracted baseline demographic characteristics and laboratory values from the Electronic Medical Record System of Xinqiao Hospital at the time of the patient’s first renal biopsy. This included demographic data such as age and gender, medical history including hypertension and history of coronary heart disease, and laboratory data such as lymphocyte count, hemoglobin, serum creatinine, blood urea nitrogen (BUN), uric acid, intact parathyroid hormone (iPTH), calcium, magnesium, phosphate, albumin, total cholesterol, low-density lipoprotein (LDL) cholesterol, high-density lipoprotein (HDL) cholesterol, triglycerides (TG), proteinuria, and pathological information. We determined estimated glomerular filtration rate (eGFR) using the Chronic Kidney Disease Epidemiology Collaboration (CKD-EPI) creatinine equation. We calculated body mass index (BMI) by dividing weight in kilograms by height in meters squared based on the obtained height and weight measurements. Additionally, we calculated the CONUT score based on three laboratory variables: serum albumin concentration, total cholesterol concentration, and total peripheral lymphocyte count. The range of CONUT scores is 0–12, where a higher score indicates worse nutritional status (Ignacio de Ulíbarri et al., 2005) (Supplementary Table S1).

### 2.3 Clinical outcomes

The study evaluated three outcomes: renal events, CVD events, and all-cause mortality, each of which was considered separately. Renal events were defined as an eGFR less than 15 mL/min/1.73 m^2^ or the need for maintenance renal replacement therapy due to irreversible deterioration of renal function, including hemodialysis, peritoneal dialysis, or kidney transplantation. CVD events were defined as the occurrence of new CVD events, such as coronary heart disease, heart failure, cerebrovascular events, and severe arrhythmia. All-cause mortality was defined as death from any cause. The study obtained clinical outcomes primarily through telephone follow-up or patient medical record reports.

### 2.4 Statistical analysis

The data analysis involved the use of SPSS (version 26.0) or R version 4.1.3. All the data used were checked for normality of distribution using the Kolmogorov-Smirnov test. Normally distributed data were expressed as mean ± standard deviation while non-normally distributed data were expressed as median (interquartile range). The differences between groups were tested using t-tests, Mann Whitney U tests, and chi-square tests. The Kaplan-Meier curve was used to compare the outcomes of the patients according to the median CONUT score. Additionally, Spearman rank correlation analysis was used to analyze the correlation between CONUT score and selected characteristics or parameters. Furthermore, the independent relationships between CONUT score and ESRD, CVD events, and all-cause mortality were investigated by univariate and multivariate Cox regression models. Hazard ratios (HRs) and 95% confidence intervals (CIs) are provided. Morevoer, subgroup analysis based on different clinical characteristics and sensitive analysis using renal pathology was also conducted to investigate the potential role of CONUT in specific populations. Furthermore, the prediction of CONUT score at different times for clinical outcomes was evaluated with time-dependent receiver operating characteristic (td-ROC) curve, and the area under the curve (AUC) was calculated for different times. It is important to note that a *p* value of < 0.05 was considered statistically significant during the analysis.

## 3 Result

### 3.1 Baseline features of the patients

Finally, a total of 336 patients with DKD was included for analysis. The mean age was 50.5 (45-59) years, and 35.1% (118) were female. Among the patients, 35.4% (119) were smokers, and 21.7% (73) had a history of coronary heart disease. A total of 70.5% (237) had hypertension. The median eGFR and proteinuria were 65.78 (39-98.70) mL/min/1.73 m^2^ and 2.26 (0.72-5.37) g/day, respectively. Then, the CONUT score was calculated with a median of 3 (IQR 1-5). Table 1 lists the baseline demographic and clinical characteristics of the study population. In addition, we also analyzed the renal pathological parameters of all patients, as show in Supplementary Table S2. Then, patients were divided into two groups based on median CONUT score: the low CONUT score group (CONUT score ≤ 3) and the high CONUT score group (CONUT score > 3). According to our findings, patients in the high CONUT group were older and more likely to have coronary heart disease, as well as higher levels of proteinuria, BUN, serum creatinine, phosphate, and LDL. They also had lower levels of hemoglobin, eGFR, albumin, and lymphocyte count. Additionally, a higher proportion of individuals in the high CONUT score group were using insulin, statins, and anti-platelet drugs. Next, we also analyzed the renal pathological parameters between the two groups and found significant differences in glomerular class, interstitial fibrosis and tubular atrophy (IFTA), interstitial inflammation, and arteriolar hyalinosis. These results suggest that patients in the high CONUT group experienced more severe renal pathological changes compared to the low CONUT score group.

**TABLE 1:** Baseline clinical characteristics of patients with diabetic kidney disease.

Clinical characteristics	Total	CONUT≤ 3	CONUT > 3	*p* value
CONUT	3 (1,5)	1 (0-1)	6 (4-7)	<0.001
Age (years)	50.5 (45-59)	50 (44,55)	53 (46-61)	0.005
Gender (Female, %)	118 (35.1)	73 (36.3)	45 (33.3)	0.574
Smoking (%)	119 (35.4)	68 (33.8)	51 (37.8)	0.458
BMI (kg/m^2^)	25.16 ± 3.33	25.38 ± 3.39	24.83 ± 3.23	0.147
Hypertension (%)	237 (70.5)	134 (66.7)	103 (76.3)	0.058
Coronary disease (%)	73 (21.7)	32 (15.9)	41 (30.4)	0.002
Pure DKD (%)	276 (82.1)	166 (82.6)	110 (81.5)	0.795
DKD combined with NDKD (%)	60 (17.9)	35 (17.4)	25 (18.5)	0.667
Medication				
Oral hypoglycemic drugs (%)	193 (57.4)	157 (78.1)	36 (26.7)	<0.001
Insulin use (%)	186 (55.4)	69 (34.3)	117 (86.7)	<0.001
RASI use (%)	262 (78.0)	159 (79.1)	103 (76.3)	0.543
Statin use(%)	156 (46.4)	84 (41.8)	72 (53.3)	0.038
Anti-platelet drug use (%)	152 (45.2)	76 (37.8)	76 (56.3)	0.01
Laboratory data				
Proteinuria (g/day)	2.26 (0.72-5.37)	1.03 (0.33-2.59)	5.35 (2.59-7.93)	<0.001
Hemoglobin (g/L)	115 (99-134.8)	125 (109-145)	105 (88-118)	<0.001
platelets (10^9/L)	200.5 (157-251.3)	187 (156-238)	210 (163-271)	0.025
eGFR (ml/min/1.73 m^2^)	65.78 (39-98.70)	75.18 (44.07-103.90)	51.23 (30.47-87.24)	<0.001
Serum creatinine (µmol/L)	109.15 (71.25-154.25)	93.5 (65.4-133.65)	129.6 (80-194.8)	<0.001
Uric acid (µmol/L)	385.40 ± 102.14	399.5 ± 103.37	364.42 ± 96.89	0.002
BUN (mmol/L)	7.15 (5.45-9.62)	6.75 (5.41-8.81)	8.09 (5.56-10.65)	0.003
Cystatin C (mg/L)	1.46 (0.99-2.12)	1.23 (0.91-1.78)	1.75 (1.28-2.53)	<0.001
Calcium (mmol/L)	2.19 (2.05-2.31)	2.27 (2.18-2.36)	2.06 (1.95-2.16)	<0.001
Magnesium (mmol/L)	0.81 (0.74-0.89)	0.82 (0.74-0.89)	0.81 (0.74-0.89)	0.772
Phosphorus (mmol/L)	1.16 (1.03-1.34)	1.13 (1.02-1.31)	1.23 (1.04-1.36)	0.017
iPTH (pg/mL)	56.3 (37.2-99.15)	53.1 (37.68-78.75)	70.5 (36-129)	0.046
Triglycerides (mmol/L)	1.69 (1.2-2.62)	1.85 (1.28-3.10)	1.47 (1.14-2.05)	<0.001
HDL (mmol/L)	1.15 (0.92-1.46)	1.07 (0.92-1.31)	1.28 (0.95-1.75)	<0.001
LDL (mmol/L)	3.1 (2.45-4.02)	2.91 (2.36-3.59)	3.41 (2.57-4.73)	<0.001
Albumin (g/L)	35.5 (27.93-42)	40.7 (16.2-44.6)	26.9 (23.4-29.9)	<0.001
Lymphocyte count (10^^9^/L)	1.6 (1.22-2.01)	1.72 (1.43-2.19)	1.35 (1.05-1.71)	<0.001
Total cholesterol (mmol/L)	5.13 (4.13-6.38)	4.88 (4.12-5.86)	5.62 (4.2-7.59)	<0.001

Note: Data are presented as mean ± SD, N (%) or median (IQR).

Abbreviations: CONUT, controlling nutritional status; BMI, body mass index; DKD, diabetic kidney disease; NDKD, non-diabetic kidney disease; RASI, renin-angiotensin system inhibitors; eGFR, estimated glomerular filtration rate; BUN, blood urea nitrogen; iPTH, intact parathyroid hormone; HDL, high-density lipoprotein; LDL, low-density lipoprotein.

### 3.2 Correlation of the CONUT score with baseline clinical characteristics

Next, we analyzed the correlations between the CONUT score and selected characteristics or laboratory variables. Spearman’s rank correlation analysis revealed that age, serum creatinine, BUN, cystatin C, LDL, and proteinuria levels were positively correlated with the CONUT score. Conversely, hemoglobin, eGFR, lymphocyte count, and albumin were negatively correlated with the CONUT score. Furthermore, with respect to renal pathological changes, the CONUT score was positively correlated with glomerular lesions (r = 0.228, *p* < 0.001), IFTA (r = 0.225, *p* < 0.001), interstitial inflammation (r = 0.222, *p* < 0.001), arteriolar hyalinosis (r = 0.149, *p* = 0.006), and arteriosclerosis (r = 0.155, *p* = 0.005) (Table 2).

**TABLE 2:** Spearman correlation between CONUT score and variables.

Variables	Correlation coefficient	*p* value
Age	0.195	< 0.001
Hemoglobin	−0.492	< 0.001
Platelets	0.07	0.204
eGFR	−0.294	< 0.001
Serum creatinine	0.264	< 0.001
Uric acid	−0.18	0.001
BUN	0.175	0.001
Cystatin C	0.35	< 0.001
Calcium	−0.657	< 0.001
Magnesium	−0.035	0.55
Phosphorus	0.103	0.061
iPTH	0.11	0.058
Triglycerides	−0.233	< 0.001
HDL	0.246	< 0.001
LDL	0.145	0.011
BMI	−0.108	0.051
Proteinuria	0.591	< 0.001
Lymphocyte count	−0.50	< 0.001
Albumin	−0.825	< 0.001
Glomerular class	0.228	< 0.001
IFTA	0.225	< 0.001
Interstitial inflammation	0.222	< 0.001
Arteriolar hyalinosis	0.149	0.006
Arteriosclerosis	0.155	0.005

Note: eGFR, estimated glomerular filtration rate; BUN, blood urea nitrogen; iPTH, intact parathyroid hormone; HDL, high-density lipoprotein; LDL, low-density lipoprotein; BMI, body mass index; IFTA, interstitial fibrosis and tubular atrophy.

### 3.3 Association between the CONUT score and endpoint events

**FIGURE 2:**
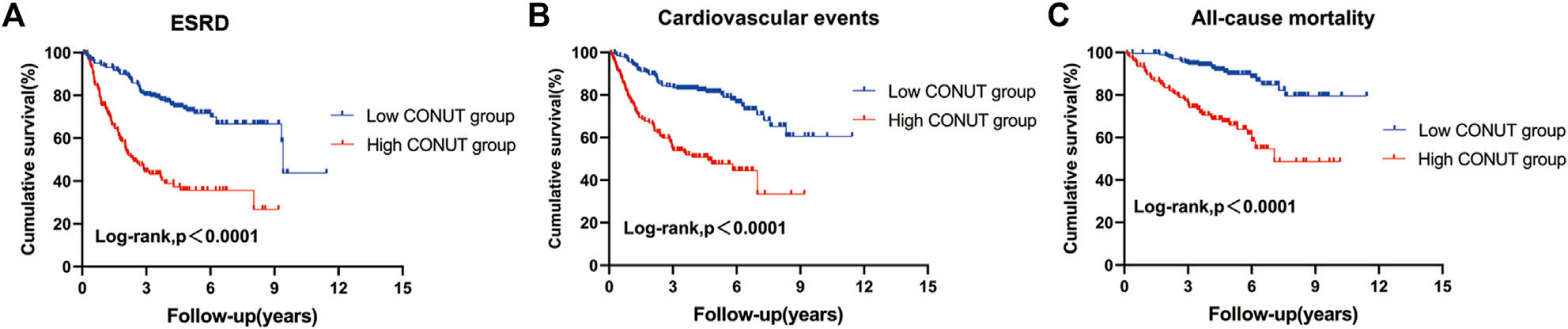
Kaplan-Meier curves for outcomes in patients with biopsy-confirmed DKD across different controlling nutritional status score (CONUT) groups. Divide patients into two groups based on the median of CONUT. the low CONUT group (CONUT≤3), the high CONUT group (CONUT>3). **(A)** End-stage renal disease (ESRD). **(B)** Cardiovascular disease. **(C)** All-cause mortality.

**FIGURE 3:**
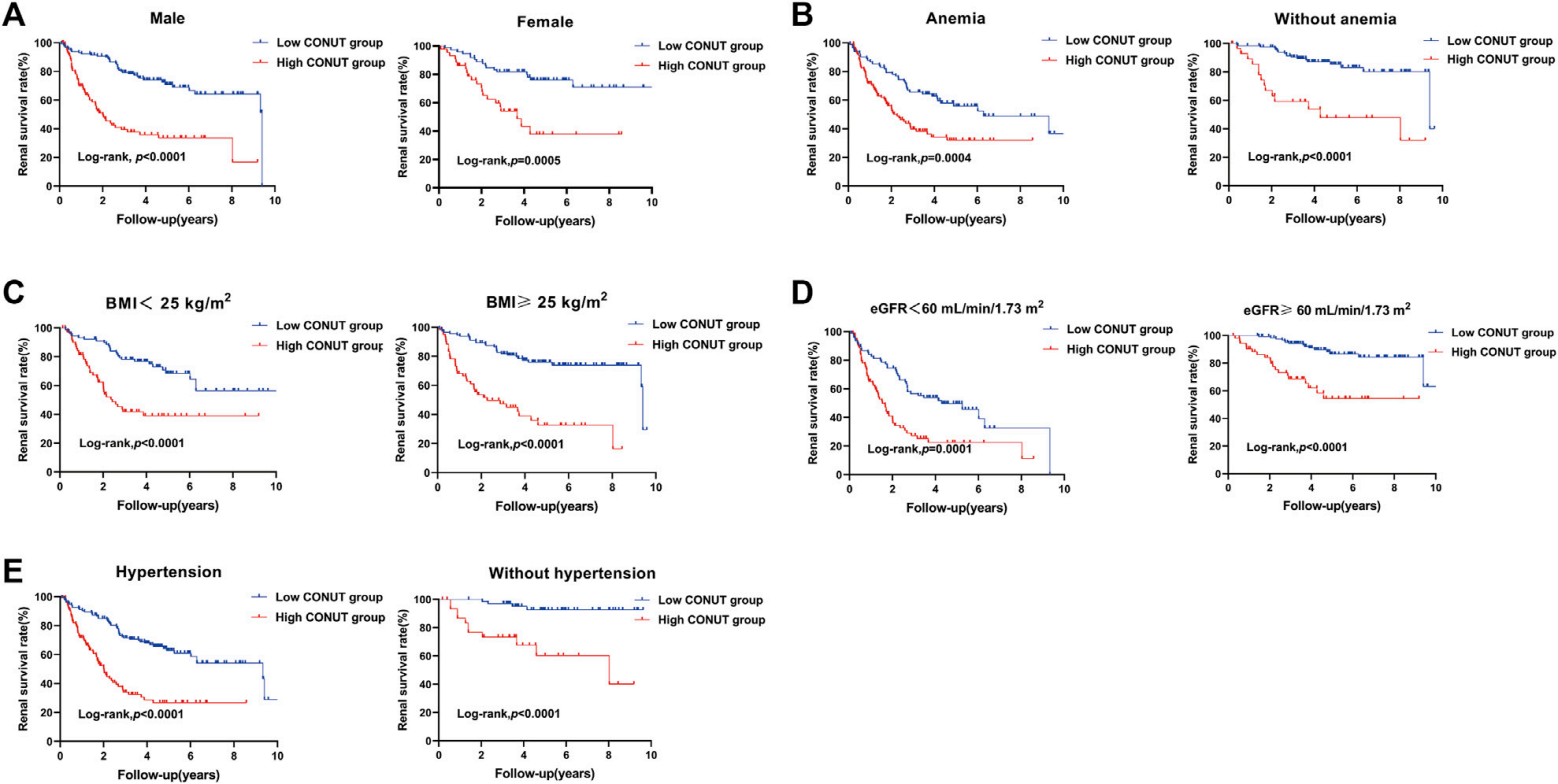
Kaplan-Meier curves for subgroup analysis in patients with biopsy-confirmed DKD with different types of clinical manifestations. Divide patients into two groups based on the median of controlling nutritional status (CONUT) score. The low CONUT group (CONUT≤3). The high CONUT group (CONUT>3). **(A)** Renal survival rate of male or female patients with different CONUT groups. **(B)** Renal survival rate of diabetic kidney disease patients with or without anemia in different CONUT groups. **(C)** Renal survival rate of diabetic kidney disease patients with serum body mass index (BMI) < 25 kg/m^2^ or ≥25 kg/m^2^ in different CONUT groups. **(D)** Renal survival rate of diabetic kidney disease patients with eGFR <60 mL/min/1.73 m^2^ or ≥60 mL/min/1.73 m^2^ in different CONUT groups. **(E)** Renal survival rate of diabetic kidney disease patients with hypertension or without hypertension in different CONUT groups.

During a median follow-up period of 5.1 years, 129 patients (38.4%) reached ESRD, while CVD events occurred among 112 individuals (33.3%), and 69 patients (20.5%) died. The Kaplan-Meier curve comparing patient’s outcomes according to median CONUT scores are shown in Figures 2A–C. The high CONUT group (CONUT score > 3) had significantly higher risk of ESRD (*p* < 0.001), CVD events (*p* < 0.01), and death (*p* < 0.01) than the low CONUT group (CONUT score ≤ 3). We also investigated the association of CONUT score with ESRD in subgroups of patients with different clinical characteristics. The results demonstrated that high CONUT group was still significantly associated with an increased risk of ESRD in males and females (Figure 3A), in patients with or without anemia (hemoglobin level < 120 g/L) (Figure 3B), in patients with BMI 25 < kg/m^2^ or not (Figure 3C), in patients with eGFR < 60 mL/min/1.73 m^2^ or not (Figure 3D), and in patients with or without hypertension (Figure 3E). Furthermore, to mitigate heterogeneity among the included patients, sensitivity analyses were conducted specifically for patients with biopsy-confirmed pure diabetic nephropathy. The Kaplan-Meier curve demonstrated that patients in the high CONUT group remained a significantly higher risk of ESRD, CVD events, and mortality (Supplementary Figure S1).

Cox proportional hazards models were used to examine the relationship between the CONUT score and renal events, CVD events, and all-cause mortality. In univariate analysis, we found that higher CONUT scores were associated with higher risk of ESRD (HR = 1.253, 95% CI 1.174-1.337, *p* < 0.001), CVD events (HR = 1.251, 95% CI 1.163-1.345, *p* < 0.001), and all-cause mortality (HR = 1.424, 95% CI 1.295-1.566, *p* < 0.001). Moreover, hypertension, hemoglobin, eGFR, iPTH, LDL, glomerular class, IFTA, and interstitial inflammation were also associated with clinical outcomes (Table 3). After adjustment for baseline age, hypertension, hemoglobin, eGFR, iPTH, LDL, glomerular class, IFTA, and interstitial inflammation in a multivariate Cox regression model, CONUT score was still an independent predictor of ESRD (HR = 1.129, 95% CI 1.037-1.228, *p* = 0.005), CVD events (HR = 1.159, 95% CI 1.057-1.271, *p* = 0.002), and all-cause mortality (HR = 1.299, 95% CI 1.143-1.478, *p* < 0.001) (Table 4).

**TABLE 3:** Univariate Cox analysis of endpoint events in patients with diabetic kidney disease.

Variable	ESRD		CVD events		Death	
	HR (95% CI)	*p* value	HR (95% CI)	*p* value	HR (95% CI)	*p* value
Age	1.00 (0.983-1.016)	0.969	1.033 (1.014-1.052)	0.001	1.055 (1.030-1.080)	< 0.001
Gender	0.693 (0.474-1.013)	0.059	0.891 (0.599-1.324)	0.567	1.215 (0.748-1.971)	0.431
BMI	0.999 (0.948-1.053)	0.974	0.996 (0.940-1.054)	0.880	0.909 (0.841-0.983)	0.016
Hypertension	4.224 (2.463-7.243)	< 0.001	2.123 (1.308-3.446)	0.002	3.512 (1.680-7.344)	0.001
Proteinuria	1.087 (1.063-1.112)	< 0.001	1.101 (1.062-1.141)	< 0.001	1.043 (0.999-1.089)	0.054
Hemoglobin	0.972 (0.964-0.980)	< 0.001	0.988 (0.981-0.996)	0.003	0.971 (0.960-0.982)	< 0.001
Platelets	1.00 (0.998-1.002)	0.927	1.001 (0.998-1.003)	0.549	1.001 (0.998-1.004)	0.483
eGFR	0.97 (0.96-0.97)	< 0.001	0.998 (0.983-0.994)	< 0.001	0.981 (0.973_0.988)	< 0.001
Uric acid	1.002 (1.00-1.003)	0.063	1.001 (0.999-1.002)	0.444	1.000 (0.998-1.003)	0.883
iPTH	1.007 (1.004-1.010)	< 0.001	1.003 (1.00-1.006)	0.028	1.004 (1.001-1.008)	0.02
TG	0.888 (0.793-0.993)	0.038	0.947 (0.856-1.047)	0.288	0.884 (0.751-1.041)	0.141
HDL	1.184 (0.816-1.716)	0.374	1.015 (0.669-1.542)	0.943	1.894 (1.225-2.927)	0.004
LDL	1.256 (1.092-1.445)	0.001	1.273 (1.094-1.480)	0.002	1.389 (1.155-1.670)	< 0.001
Glomerular class	1.883 (1.594-2.224)	< 0.001	1.481 (1.253-1.752)	< 0.001	1.363 (1.104-1.683)	0.004
IFTA	2.475 (1.994-3.071)	< 0.001	1.469 (1.198-1.801)	< 0.001	1.422 (1.097-1.844)	0.008
Interstitial inflammation	3.387 (2.422-4.735)	< 0.001	1.705 (1.252-2.320)	0.001	1.55 (1.055-1.278)	0.026
Arteriolar hyalinosis	3.215 (1.949-5.304)	< 0.001	1.477(0.994-2.195)	0.054	1.898 (1.069-3.371)	0.029
Arteriosclerosis	1.561 (1.213-2.009)	0.001	1.356 (1.043-1.762)	0.023	1.299 (0.934-1.806)	0.120
CONUT	1.253 (1.174-1.337)	< 0.001	1.251 (1.163-1.345)	< 0.001	1.424 (1.295-1.566)	< 0.001

Note: HR, hazards ration; CI, confidence interval; CONUT, ontrolling nutritional status; ESRD, end-stage renal disease; CVD: cardiovascular; BMI, body mass index; eGFR, estimated glomerular filtration rate; iPTH, intact parathyroid hormone; TG, triglycerides; HDL, high-density lipoprotein; LDL, low-density lipoprotein; IFTA, interstitial fibrosis and tubular atrophy.

**TABLE 4:** Multivariate Cox analysis of endpoint events in patients with diabetic kidney disease.

	Model 1		Model 2		Model 3	
	HR (95% CI)	*p* value	HR (95% CI)	*p* value	HR (95% CI)	*p* value
ESRD						
CONUT score	1.270 (1.188-1.358)	< 0.001	1.193 (1.106-1.287)	< 0.001	1.129 (1.037-1.228)	0.005
CVD events						
CONUT score	1.229 (1.141-1.324)	< 0.001	1.226 (1.131-1.329)	< 0.001	1.159 (1.057-1.271)	0.002
Death						
CONUT score	1.379 (1.251-1.521)	< 0.001	1.335 (1.201-1.484)	< 0.001	1.299 (1.143-1.478)	< 0.001

Note: CI, confidence interval; CONUT, controlling nutritional status score; ESRD, end-stage renal disease; CVD: cardiovascular disease; eGFR, estimated glomerular filtration rate; iPTH, intact parathyroid hormone; LDL, low-density lipoprotein; RASI, renin-angiotensin system inhibitors; IFTA, interstitial fibrosis and tubular atrophy.

Model 1: adjusted age.

Model 2: adjusted age, hypertension and hemoglobin.

Model 3: adjusted age, hypertension, hemoglobin, eGFR, iPTH, LDL, glomerular class, IFTA, interstitial inflammation.

### 3.4 The CONUT score and the prediction of clinical outcomes

**FIGURE 4:**
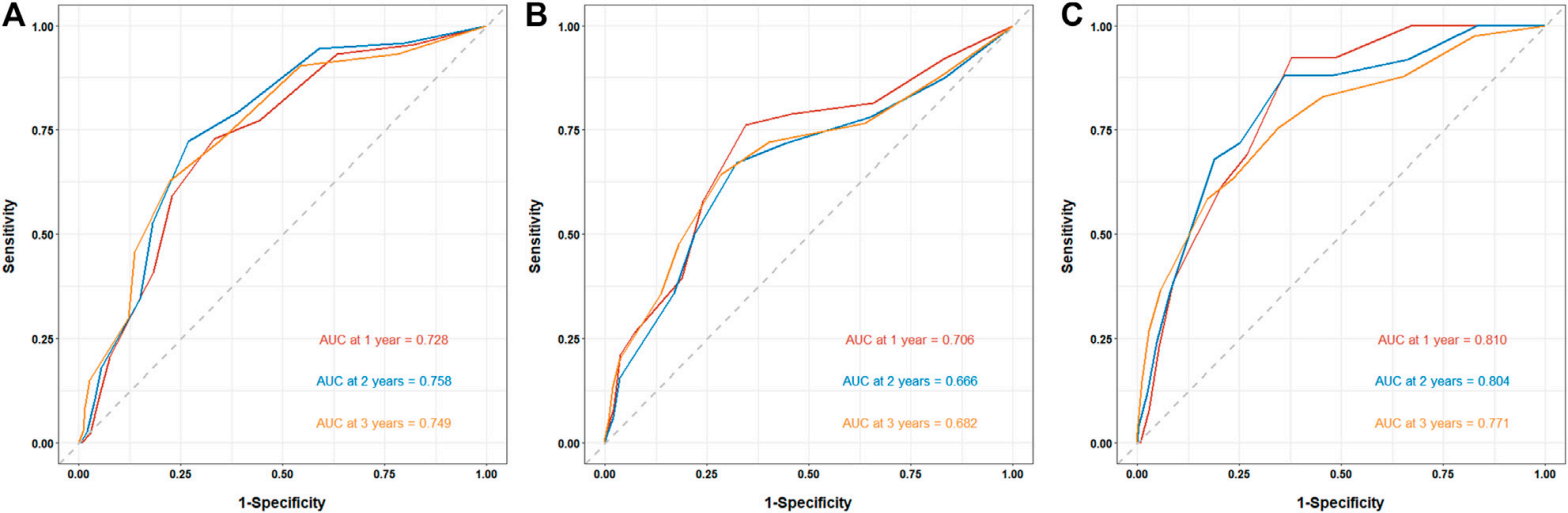
The prediction of CONUT score for clinical outcomes in patients with biopsy-confirmed DKD. The prediction of clinical outcomes by the CONUT score was evaluated using the time-dependent receiver operating characteristic (td-ROC) of the subjects. **(A)** Prediction of ESRD by CONUT score. **(B)** Prediction of CVD events by CONUT score. **(C)** Prediction of all-cause mortality by CONUT score.

The prediction of clinical outcomes by the CONUT score was evaluated using the time-dependent receiver operating characteristic (td-ROC) of the subjects. For the prediction of ESRD, the area under curve (AUC) was 0.728 at 1 year, 0.758 at 2 years, and 0.749 at 3 years, respectively (Figure 4A). For the prediction of CVD events, the AUC was 0.706 at 1 year, 0.666 at 2 years, and 0.682 at 3 years, respectively (Figure 4B). And the prediction of all-cause mortality, the AUC was 0.810 at 1 year, 0.804 at 2 years, and 0.771 at 3 years, respectively (Figure 4C).

## 4 Discussion

In this study, we investigated the relationship between the CONUT score and outcomes in patients with diabetic kidney disease. Our study is the first to show that the CONUT score can be used as a clinical predictor of ESRD, CVD events, and all-cause mortality in patients with diabetic kidney disease.

In our study, a high CONUT score was found to be associated with an increased risk of ESRD in patients with diabetic kidney disease, which is the most common complication in these patients. Research has suggested that immune inflammation and nutritional status may be closely related to the progression of renal function in diabetic kidney disease patients (Zhang et al., 2022; Liu et al., 2023). Among the components of CONUT score, albumin serves as a cornerstone for nutritional assessment. Zhang et al. have confirmed that hypoalbuminemia is associated with the progression of ESRD in patients with diabetic kidney disease (Zhang et al., 2019). Lymphocytes in the CONUT score, which are immune cells involved in mediating the inflammatory response. A previous retrospective study showed that the platelet-lymphocyte ratio (PLR) is an independent risk factor for progression to ESRD in patients with diabetic kidney disease (Duan et al., 2021), suggesting that low lymphocyte counts may lead to ESRD. In our study, Cox regression analysis showed that high CONUT scores (indicating low albumin and lymphocyte counts) were significantly associated with an increased risk of diabetic kidney disease progression to ESRD, which is consistent with the aforementioned research results. In addition, there is evidence that the neutrophil-lymphocyte ratio (NLR) is significantly associated with decreased eGFR and histopathological changes in diabetic nephropathy patients (Zhang et al., 2019a). Similarly, we found that high CONUT scores were associated with lower eGFR and more severe pathological damage. To more accurately assess the effect of CONUT scores on ESRD, we performed different subgroup analyses, and the results further showed that the higher CONUT score, the higher the risk of progression to ESRD in different subgroups of diabetic kidney disease patients. Therefore, the CONUT score, as a combined index of nutrition, immunity, and inflammation, may be a more comprehensive predictor of diabetic kidney disease progression to ESRD. Routine assessment of the CONUT score can assist physicians in developing appropriate treatment plans for patients with diabetic kidney disease, thereby slowing their kidney progression.

We observed a significant correlation between CONUT score and the incidence of CVD events in diabetic kidney disease patients. The risk of CVD is high in patients with diabetic kidney disease, which is a major cause of mortality (Valmadrid et al., 2000; Gerstein et al., 2001). The potential mechanisms underlying the association between the CONUT score and CVD events are complex and multifaceted. but each component of the CONUT score is related to the CVD events. High cholesterol is a conventional risk factor for CVD in the general population. However, in dialysis patients, low levels of cholesterol are a high-risk factor for CVD events (Kalantar-Zadeh et al., 2003). Additionally, studies have shown that low levels of cholesterol are associated with CVD events in patients with chronic heart failure (Nakagomi et al., 2014). The reason for this lipid paradox may be that the inflammatory or malnutrition state of the organism leads to a disturbance in lipid metabolism, which increases the risk of adverse events (Liu et al., 2004). Also, lymphocytes, as immune cells, are involved in systemic inflammatory responses, and clinical and animal studies have shown that low lymphocyte counts promote atherosclerosis formation (Núñez et al., 2011). Finally, the synthesis of albumin is influenced by chronic inflammation and malnutrition, and lower levels of albumin may be a marker of continuous arterial injury, as well as the progression of atherosclerosis and thrombosis (Kuller et al., 1991). A clinical study has also shown that lower serum albumin levels are associated with a higher risk of CVD events in patients with diabetic kidney disease (Anavekar et al., 2004). Therefore, the CONUT score, as a combination of the above three indicators, may be a reliable biomarker for identifying high-risk CVD patients. Early assessment of the CONUT score can provide a preliminary understanding of the nutritional, immune, inflammatory, and lipid metabolism status of patients and provide a reference for clinical management.

In the present study, a higher CONUT score was related to higher all-cause mortality. Nutritional status has a strong correlation with mortality in patients diagnosed with diabetic kidney disease (Tauchi et al., 2020). A previous cohort study comprising 2,720 patients indicated that nutritional status assessed by prognostic nutritional index (PNI) was related to all-cause mortality in patients with diabetic kidney disease (Zhang et al., 2023). In line with these findings, our study revealed that higher CONUT scores implied poorer nutritional status and increased risk of all-cause mortality, thus further emphasizing the importance of nutritional status in the development of all-cause mortality in diabetic kidney disease patients. In addition, anemia and BMI are reliable indicators for assessing the nutritional status of patients, with previous studies highlighting that patients with anemia have a higher risk of mortality (Vlagopoulos et al., 2005), and low weight diabetic kidney disease patients, characterized by BMI (<18.5 kg/m^2^), also have a higher risk of mortality (Yokomichi et al., 2021). Consistent with previous studies, our univariate Cox analysis demonstrated that patients with lower hemoglobin and BMI had a higher risk of all-cause mortality. In general, the CONUT score, serving as a nutritional assessment index, effectively reflects the immune-nutritional status of patients. By identifying patients who are prone to malnutrition, early nutritional support can be administered, thereby reducing the incidence of mortality.

## 5 Limitation

Some limitations in this study deserve our attention. First, we were a single-center study involving a small number of people and only included patients with renal biopsy-proven DKD, which may have had selective bias. Moreover, there was heterogeneity in the included patients with different types of treatment drugs, which may have an impact on the occurrence of endpoint events. Second, we only assessed the CONUT score at the time of patient enrollment without investigating the prognostic impact of CONUT score changes over time in patients with diabetic kidney disease. Third, the validity of nutritional status assessed by CONUT score remains uncertain because of the lack of comparison with comprehensive nutritional assessment tools such as the Subjective Global Assessment (SGA) and Mini Nutritional Assessment (MNA).

## 6 Conclusion

In conclusion, the CONUT score serves as a simple and easily accessible biomarker for predicting progression to ESRD, CVD events and all-cause mortality in patients with diabetic kidney disease. Early assessment of the CONUT score can identify people at high risk of diabetic kidney disease and is important for reducing adverse clinical outcomes.

## Data Availability

The raw data supporting the conclusion of this article will be made available by the authors, without undue reservation.

## Supplementary material

Supplementary Figure S1 Kaplan-Meier curves for **(A)** End-stage renal disease (ESRD), **(B)** Cardiovascular disease (CVD) events and **(C)** All-cause mortality in patients with biopsy-confirmed pure diabetic nephropathy (DN) across different controlling nutritional status score (CONUT) groups. Divide patients into two groups based on the median of CONUT. The low CONUT group (CONUT≤3), the high CONUT group.
