# Supplementary figures and images for: Controlling nutritional status score is associated with renal progression, cardiovascular events, and all-cause mortality in biopsy-proved diabetic kidney disease
Source: Front Physiol. 2023 Aug 7;14:1231448. doi: 10.3389/fphys.2023.1231448 (PMC10440377; doi:10.3389/fphys.2023.1231448)

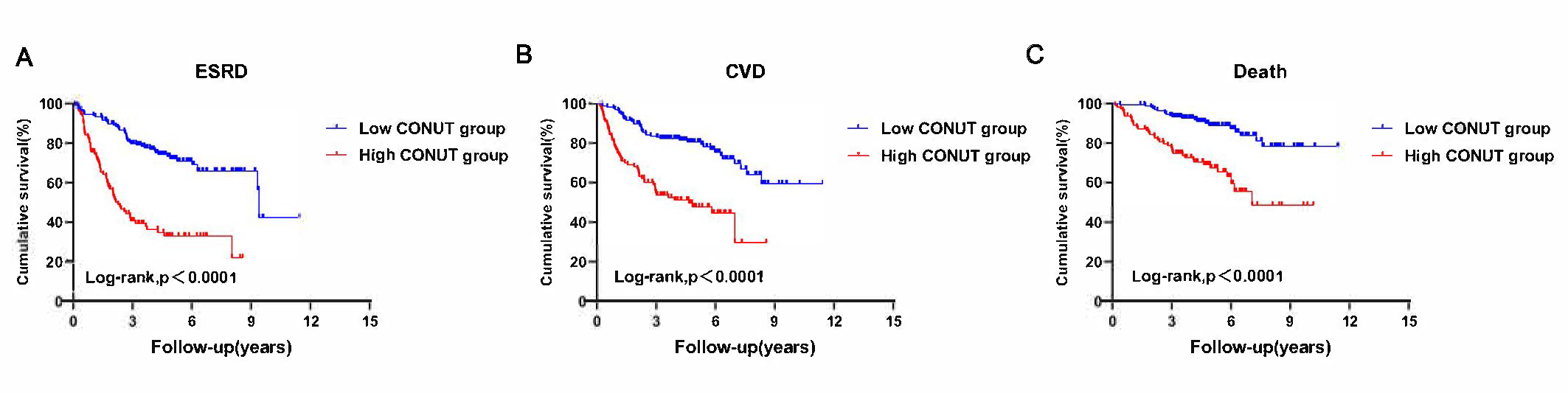

Supplement: Supplementary file 1 [file Image1.TIFF]
