# Supplementary material for: Controlling nutritional status score is associated with renal progression, cardiovascular events, and all-cause mortality in biopsy-proved diabetic kidney disease
Source: Front Physiol. 2023 Aug 7;14:1231448. doi: 10.3389/fphys.2023.1231448 (PMC10440377; doi:10.3389/fphys.2023.1231448)
Supplement: Supplementary file 2 [file Table1.DOCX]

Supplementary Material

**Controlling Nutritional Status (CONUT) score is associated with renal progression, cardiovascular events, and all-cause mortality in biopsy-proved diabetic nephropathy**

**Qingyu Huo^1^, Ting He^1^, Jiachuan Xiong^1^*, Jinghong Zhao^1^***

*** Correspondence:** Corresponding author: Jinghong Zhao, M.D., Ph.D. Address: Department of Nephrology, the Key Laboratory for the Prevention and Treatment of Chronic Kidney Disease of Chongqing, Chongqing Clinical Research Center of Kidney and Urology Diseases, Xinqiao Hospital, Army Medical University (Third Military Medical University), Chongqing, China, 400037. Tel: +86-23-68774321, Fax: +86-23-68774321, E-mail: zhaojh@tmmu.edu.cn. Alternatively, Jiachuan Xiong, Address: Department of Nephrology, the Key Laboratory for the Prevention and Treatment of Chronic Kidney Disease of Chongqing, Chongqing Clinical Research Center of Kidney and Urology Diseases, Xinqiao Hospital, Army Medical University (Third Military Medical University), Chongqing, China, 400037. Tel: +86-23-68774428, Fax: +86-23-68774428, E-mail: xiongjc@tmmu.edu.cn.

# Supplementary Tables

| **Supplementary Table 1. Parameters for assessment of the CONUT Score.** | | | | |
| --- | --- | --- | --- | --- |
| Parameter | Score | | | |
| Serum albumin (g/ml) | ≥ 3.5 | 3.0-3.49 | 2.50-2.99 | 2.50 |
| Albumin score | 0 | 2 | 4 | 6 |
| Total cholesterol (mg/dl) | ≥ 180 | 140-179 | 100-139 | < 100 |
| Cholesterol score | 0 | 1 | 2 | 3 |
| Lymphocytes (count/ml) | ≥ 1600 | 1200-1599 | 800-1199 | < 800 |
| Lymphocytes score | 0 | 1 | 2 | 3 |

| **Supplementary Table 2. Pathologic features of patients with different CONUT score** | | | | |
| --- | --- | --- | --- | --- |
| **Pathological Lesions** | **All** | **Low group**  **(CONUT≤3)** | **High group**  **(CONUT＞3)** | ***P* Value** |
| **Glomerular class** |  |  |  | ＜0.001 |
| I (%) | 33 (9.80) | 23 (11.40) | 10 (7.40) |  |
| IIa (%) | 84 (25.00) | 62 (30.80) | 22 (16.30) |  |
| IIb (%) | 63 (18.80) | 43 (21.40) | 20 (14.80) |  |
| III (%) | 119 (35.40) | 55 (27.40) | 64 (47.40) |  |
| IV (%) | 37 (11.00) | 18 (9.00) | 19 (14.10) |  |
| **IFTA** |  |  |  | 0.02 |
| 0 (%) | 27 (8.00) | 18 (9.00) | 9 (6.70) |  |
| 1 (%) | 114 (33.90) | 81 (40.30) | 33 (24.40) |  |
| 2 (%) | 100 (29.80) | 59 (29.40) | 41 (30.40) |  |
| 3 (%) | 95 (28.30) | 43 (21.40) | 52 (38.50) |  |
| **Interstitial inflammation** |  |  |  | 0.01 |
| 0 (%) | 35 (10.40) | 22 (10.90) | 13 (9.60) |  |
| 1 (%) | 157 (46.70) | 109 (54.20) | 48 (35.60) |  |
| 2 (%) | 144 (42.90) | 70 (34.80) | 74 (54.80) |  |
| **Arteriolar hyalinosis** |  |  |  | 0.012 |
| 0 (%) | 12 (3.6) | 8 (4.00) | 4 (3.00) |  |
| 1 (%) | 85 (25.3) | 62 (30.8) | 23 (17.00) |  |
| 2 (%) | 23 (71.1) | 131 (65.2) | 108 (80.00) |  |
| **Arteriosclerosis** |  |  |  | 0.127 |
| 0 (%) | 69 (20.50) | 46 (22.90) | 23 (17.00) |  |
| 1 (%) | 153 (45.50) | 95 (47.30) | 58 (43.00) |  |
| 2 (%) | 114 (33.90) | 60 (29.90) | 54 (40.00) |  |
| Note : Data are presented as N (%). P value for the chi-squared test.  Abbreviations: IFTA, interstitial fibrosis and tubular atrophy. | | | | |
